# Bayesian Comparison of Neurovascular Coupling Models Using EEG-fMRI

**DOI:** 10.1371/journal.pcbi.1002070

**Published:** 2011-06-16

**Authors:** Maria J. Rosa, James M. Kilner, Will D. Penny

**Affiliations:** Wellcome Trust Centre for Neuroimaging, Institute of Neurology, University College London, London, United Kindgom; University of Oxford, United Kingdom

## Abstract

Functional magnetic resonance imaging (fMRI), with blood oxygenation level-dependent (BOLD) contrast, is a widely used technique for studying the human brain. However, it is an indirect measure of underlying neuronal activity and the processes that link this activity to BOLD signals are still a topic of much debate. In order to relate findings from fMRI research to other measures of neuronal activity it is vital to understand the underlying neurovascular coupling mechanism. Currently, there is no consensus on the relative roles of synaptic and spiking activity in the generation of the BOLD response. Here we designed a modelling framework to investigate different neurovascular coupling mechanisms. We use Electroencephalographic (EEG) and fMRI data from a visual stimulation task together with biophysically informed mathematical models describing how neuronal activity generates the BOLD signals. These models allow us to non-invasively infer the degree of local synaptic and spiking activity in the healthy human brain. In addition, we use Bayesian model comparison to decide between neurovascular coupling mechanisms. We show that the BOLD signal is dependent upon both the synaptic and spiking activity but that the relative contributions of these two inputs are dependent upon the underlying neuronal firing rate. When the underlying neuronal firing is low then the BOLD response is best explained by synaptic activity. However, when the neuronal firing rate is high then both synaptic and spiking activity are required to explain the BOLD signal.

## Author Summary

Functional magnetic resonance imaging (fMRI), with blood oxygenation level-dependent (BOLD) contrast, is a widely used technique for studying the human brain. However, the relationship between neuronal activity and blood flow, the basis of fMRI, is still under much debate. A growing body of evidence from animal studies suggests that fMRI signals are more closely coupled to synaptic input activity than to the spiking output of a neuronal population. However, data from neurosurgical patients does not seem to support this view and this hypothesis hasn't yet been tested in the healthy human brain. Here we design a powerful and efficient modelling framework that can be used to non-invasively compare different biologically plausible hypotheses of neurovascular coupling. We use this framework to explore the contribution of these two aspects of neuronal activity (synaptic and spiking) to the generation of hemodynamic signals in human visual cortex, with Electroencephalographic (EEG)-fMRI data. Our results provide preliminary evidence that depending on the frequency of the visual stimulus and underlying firing rate, fMRI relates closer to synaptic activity (low-frequencies) or to both synaptic and spiking activities (high-frequencies).

## Introduction

Functional magnetic resonance imaging (fMRI) is an extensively employed neuroimaging technique that allows the non-invasive recordings from human brain of neuronal activity with relatively high spatial resolution. However, the blood oxygenation level-dependent (BOLD) contrast on which fMRI is based is only an indirect measure of this activity. The processes that link the underlying neuronal activity to the BOLD signals are still a topic of much debate. In particular, there is no consensus on the relative roles of synaptic and spiking activity in the generation of BOLD signals. In order to relate findings from fMRI research to other measures of neuronal activity it is important to understand the underlying neurovascular coupling mechanism [1].

Most of our present knowledge about neurovascular coupling comes from animal experiments. These studies have combined hemodynamic measures such as cerebral blood flow (CBF), with electrical measurements such as local field potentials (LFPs) and single/multi-unit activity (S/MUA). LFPs correspond primarily to weighted averages of synchronised dendro-somatic components of synaptic signals in a neuronal population, whilst S/MUA measures the action potentials of a single cell or population of cells, respectively [2].

In a pioneering study [3], found, in monkey visual cortex, that although both LFPs and MUA correlate with the BOLD response, this response could be predicted more accurately from LFPs. This result has been confirmed in awake animals [4]. On the other hand, [5], [6] and [7] found strong positive correlations between blood flow and spiking activity. More recently, [8], [9] and [10] have shown that when synaptic and spiking activity is uncoupled (by drug injection in [8], [10] and using a stimulus that elicits only synaptic activity in [9]), changes in CBF do not reflect underlying spiking activity and relate closer to LFPs.

This growing body of evidence (Table 1) therefore supports the hypothesis that BOLD signals are more closely coupled to synaptic input and processing activity than to the output spikes of a population of neurons. In addition, this work (Table 1) provides support to a growing consensus in which the BOLD signal is thought to result from pre-synaptic activity and the release of neurotransmitters, in particular glutamate [11], as well as vasodilatory substances, such as nitric oxide [12], [13] and [14]. An increase in pre-synaptic activity and concomitant release of glutamate induces fluctuations in transmembrane potential at the post-synaptic neuron, and these fluctuations are measured with LFPs. This activity is also thought to be responsible for triggering the release of vasodilatory agents to the extracellular medium, which induce changes in blood flow and consequently the BOLD response [11].

**Table 1:** Main findings of previous animal studies on neurovascular coupling.

Reference	Paradigm	Main findings	Brain regions	Species	Signals
[3]	Visual (rotating checkerboard)	LFP (40–130 Hz) better predictor of BOLD than MUA (300–1.5 kHz)	V1	Monkey	BOLD, LFP, MUA
[4]	Visual (rotating checkerboard)	BOLDÕs variance best explained by LFP (20–60 Hz)	V1	Monkey (awake)	BOLD, LFP, MUA, SUA
[5]	Visual (moving dots; changing coherence)	BOLD contrast in human V5 isproportional to SUA in monkey V5	V5	Monkey/Human	BOLD, SUA
[6]	Visual (changing contrast)	BOLD in human V1 is proportional to SUA in monkey V1	V1	Monkey/Human	BOLD, SUA
[8]	Resting-state	Drug induced increase in Purkinje cell spike activity was not sufficient to raise blood flow above baseline	Cerebellum	Rat	CBF, SUA
[9]	Visual (sine-wave gratings, 1–20 Hz)	Correlation between BOLD and LFPs in the absence of spiking activity (suppressed by the stimulus)	V1	Cat	LFP, MUA, 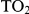
[10]	Visual (rotating checkerboard)	Injected neuromodulator BP554 induces hyperpolarization of efferent membrane, reducing MUA (800–3 k Hz) without affecting either LFP (24–90 Hz) or BOLD activity	V1	Monkey	BOLD, LFP, MUA
[70]	Visual (sinewave gratings, natural movies and pink pixel noise)	Agreement between BOLD and LFP (in terms of 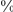 of recording sites) depends on LFP frequency. Best agreement between 20 and 50 Hz. Poorer agreement for MUA	Visual cortex (17,18,19 and 21a)	Cat	BOLD, LFP, MUA
[71]	Visual	BOLD correlates better with gamma-band LFP	Visual cortex	Cat	BOLD, LFP, MUA
[72]	Perceptual suppresion	Only BOLD and low-Hz LFP (not high-Hz LFP or spikes) significantly decreased during perceptual suppression	V1	Monkey (awake)	BOLD, LFP, Spikes
[68]	Whisker pad stimulation	Deep layer negative BOLD, adjacent to layers of positive BOLD, associated with reductions in MUA	Somato-sensory cortex	Rat	BOLD, LFP, MUA, OHb, dHb, CBV
[64]	Optical stimulus	Negative BOLD signal caused by optically driving genetically modified inhibitory cells	Motor cortex	Rat	Optogenetics

S/MUA refers to single/multi-unit activity. CBF refers to cerebral blood flow; 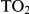 to tissue oxygenation concentration; OHb, dHb, CBV to oxy and deoxy-Hemoglobin and cerebral blood volume, respectively.

However, when it comes to the human brain the number of studies directly addressing the question of how BOLD relates to synaptic versus spiking activity is relatively smaller (Table 1 and 2), and the data in these studies comes exclusively from neurosurgical patients, whose physiology may be compromised (Table 2). Of the few such studies [15], observe significant correlations between BOLD signals and both synaptic and spiking signals in auditory cortex, whilst [16] found no correlation between BOLD signals and neuronal firing in the hippocampal area.

**Table 2:** Main findings of previous human studies on neurovascular coupling.

Reference	Paradigm	Main findings	Brain regions	Species	Signals
[15]	Movie segment	Significant correlation between patients predicted BOLD signals from SUA and signals measure in healthy subjects	Auditory cortex	Human (patients)	BOLD, LFP, SUA
[16]	Spatial navigation in virtual environment	Correlation between the BOLD signal andtheta-band activity; no significant correlation with MUA/SUA	Hippocampal areas	Human (patients)	BOLD, LFP, MUA, SUA
[18]–[21]	Resting-state	Reductions in alpha power correlate with increases in BOLD	Occipital cortex	Human (healthy)	BOLD, EEG
[21]	Semantic decision task	Close spatial correspondence between BOLD activation regions and gamma-ECoG sites	Temporal and sulcal cortex and insula	Human (patients)	BOLD, ECoG
[34]	Visual (flickering checkerboard 4–60 Hz)	Root-mean squared frequency explains more BOLD activity than the total spectral power or any linear combination of frequency-bands	Visual cortex	Human (healthy)	BOLD, EEG
[73]	Movie segments	Gamma-LFP coupled well to BOLD; coupling for SUA highly variable	Auditory cortex	Human (patients)	BOLD, LFP, SUA
[74]	Wakefulness (AW), slow-wave and rapid-eye-movement sleep (REM)	State-invariant significant structural correlation between BOLD and slow cortical potentials ( 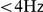 ). Gamma band potentials only correlate with BOLD during AW and REM	Sensori-motor cortex	Human (patients)	BOLD, ECoG
[75]	Resting-state	BOLD response is negatively correlated with GABA concentration and gamma oscillation frequency	Visual cortex	Human (healthy)	MEG, GABA concentration

ECoG refers to Electrocorticography.

The link between neuronal activity and the BOLD response has not only been investigated at a microscopic level, using invasive co-localised recordings, but also at a macroscopic scale using fMRI and Electroencephalography (EEG). EEG (and Magnetoencephalography (MEG)), are well established non-invasive techniques that are well suited to studying neuronal activity since they provide direct (not confounded by the hemodynamic response) measurement of post-synaptic potentials (magnetic fields) in cortical pyramidal cell populations with high temporal resolution [17]. Studies using both EEG and fMRI in humans have focused on correlations between BOLD signals and oscillatory EEG power measured in different frequency bands. For example, [18], [19] and [20] have shown that reductions in ongoing-scalp EEG alpha power (8–13 Hz) correlate with increases in BOLD activity in human occipital cortex. Using intra-cranial recordings in epileptic patients [21], have found a close spatial correspondence between regions of fMRI activation and sites showing EEG energy variation in the gamma band (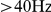). The main conclusion of this body of work is that increases in EEG frequency are associated with increases in BOLD signal. Even though these studies do not address our question (input versus output) directly, they seem to point in the direction of the biological hypothesis constructed from animal evidence (see above): increases in pre-synaptic activity, decrease effective membrane time-constants and result in faster oscillatory dynamics; at the same time more neurotransmitters are released (e.g. glutamate), which lead to increases in BOLD signal [14].

Here we design a powerful and efficient modelling framework to explicitly investigate competing hypotheses for the relationship between neuronal activity and the BOLD response in the healthy human brain. We use this framework to explore the relative contribution of synaptic and spiking activity to the generation of fMRI signals in visual cortex.

The participation of healthy subjects prohibits the use of invasive electrophysiological measures. Therefore we use a mathematical modelling framework that allows us to non-invasively infer the degree of local synaptic and spiking activity, together with EEG-fMRI data, in which subjects were exposed to a reversing checkerboard of varying frequencies. This is similar in spirit to the use of ‘virtual electrodes’ in EEG analysis [22], but provides more specific biophysical information. This framework consists of a biophysically informed forward model from neuronal activity to the observed EEG and fMRI signals.

Models linking neuronal activity to EEG/MEG signals have been proposed by [23], [24] and [25], to mention a few. These models usually use one or two state variables to represent the mean electrical activity of neuronal populations at the macro-column level, and are referred to as neural mass models [26]. Models linking ‘neuronal activity’ to BOLD signals include the metabolic models proposed by [27], [28] and the Balloon model, proposed by [29]. The Balloon model describes how evoked changes in blood flow are transformed into the BOLD response and has been extended by [30], who introduced a blood flow-inducing signal relating ‘neuronal activity’ and CBF and by [31], where different metabolic pathways have been proposed for supporting excitatory and inhibitory synaptic activity. In the above work ‘neuronal activity’ is usually not explicitly modelled and often corresponds to the stimulus input functions.

Models linking a common underlying neuronal substratum to both EEG and fMRI signals have also been developed [32]. Some models are phenomenologically motivated, such as the ‘Heuristic’ proposed by [33]. This model aims to explain empirical results which relate frequency-specific power changes in EEG with fMRI signals and predicts that increases in the BOLD contrast reflect increases in the Root Mean Squared (RMS) frequency of EEG. We have validated these predictions in previous work [34] using simultaneous EEG-fMRI data in humans with a visual flicker stimulation task. As predicted by [33], the RMS frequency significantly explained more BOLD activity than the total time-varying spectral power or any linear combination of frequency-band amplitude modulations (e.g. alpha or gamma power).

Biophysically motivated models include [35]–[37]. Most of these theoretical frameworks combine the neural mass model approach for EEG with the Balloon model for fMRI, but the coupling between neuronal activity and blood flow differs from model to model. For instance [35], propose that the squared post-synaptic membrane potential from both excitatory and inhibitory cells from a cortical area drives increases in cerebral blood flow, whilst [37] consider all the incoming action potentials from populations within and outside the voxel to be the input to the BOLD response. In [36] this input is proportional to the total concentration of nitric oxide (NO) synthesised by neurons in the cortical unit. The parameters of this model have been estimated using EEG-fMRI data from the visual cortex of one subject exposed to a reversing checkerboard with varying frequency [38].

Despite these theoretical efforts, the existing modelling frameworks have not yet been used in conjunction with real electrophysiological and hemodynamic data to compare different neurovascular coupling mechanisms, although important steps in this direction have been taken by [36], [39]. In [39], the authors have compared different models to investigate the role of excitatory and inhibitory activity in the generation of BOLD signals, using fMRI data from one subject. They found BOLD signals to be best explained by excitatory activity alone.

Here we use the forward model proposed by [36] and embed it within a Bayesian framework. Using EEG and fMRI data in combination with Bayesian inference allows us to estimate the underlying synaptic and spiking activity, along with other biophysical model parameters. These quantities are computed using the variational Laplace method described in [40]. This optimisation scheme has been successfully applied to other input-state-output systems, such as [41], [42].

However, inverting generative models using multi-modality datasets, can be a technically demanding task, if the temporal characteristics of the datasets are very different, which is the case for EEG-fMRI data. Here we develop a computationally efficient scheme for model inversion. Instead of inverting the model in a single (computationally demanding) step we adopt a ‘multi-step inversion’ approach. This approach is based on partitioning model inversion into multiple, independent and computationally efficient steps that are motivated by the time-scales of data involved. This is a general procedure that can be used with other datasets and in other multimodal studies, such as with MEG-fMRI or LFP-fMRI data.

Finally, once equiped with this mathematical and computational framework we posit models embodying different hypotheses about neurovascular coupling and adjudicate between them using Bayesian model evidence [43]. We compare three models. The first assumes that blood flow depends on the amount of vasodilatory substances (e.g. nitric oxide) released as a result of synaptic activity (*synaptic input model*), as proposed by [36]. The second assumes blood flow is driven by the firing rate of pyramidal cells from the same unit (*spiking output model*). These hypotheses are then compared against a third model where both these quantities contribute to the BOLD response (*mixture model*). In the long term, we anticipate that this modelling framework will be used to test neurovascular coupling hypotheses in a variety of experimental contexts with a range of subject cohorts.

## Materials and Methods

### Local electro-vascular (LEV) model

**Figure 1:**
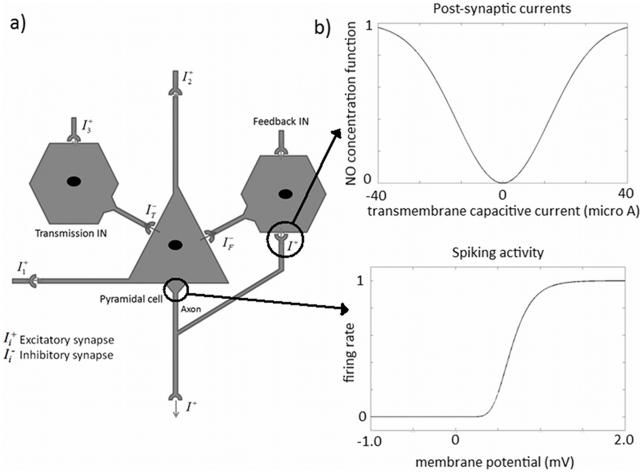
Local electro-vascular model: cortical unit. a) The unit comprises three subpopulations of cells, two layer IV GABAergic interneurons and a layer V pyramidal cell. The unit receives input from cortical or thalamic connections, 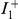, 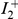 and 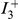, whilst its output is the firing rate of layer V pyramidal cells, 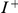; b) Non-linear function of the transmembrane capacitive currents used to calculate the NO concentration. This function is symmetric because both positive and negative currents increase the amount of NO released. This function is used in the synaptic input coupling model. c) Sigmoid function from membrane potential to firing rate. This function is used as the input to the vascular equations in the spiking output model.

We use a realistic biophysical model, proposed by [36], of how electrical and vascular dynamics are generated within a cortical unit. The unit comprises three subpopulations of cells: two layer IV GABAergic interneuron populations (the transmission and feedback interneurons (INs)) and a layer V pyramidal cell (PC) population (Figure 1(a)). Interneurons are modelled as single compartment neurons, whilst the pyramidal cell has three compartments (soma, basal and apical tuft dendrites). Here we briefly describe the forward model. A summary of all the equations and parameters of the model can be found in Text S1. For a more detailed description please consult the original work [36].

### Neural mass model

A neural mass model (NMM) characterises the population dynamics of electrical states such as the membrane potentials in the somas of the neurons and electric currents flowing in the neuropil. This modelling framework is appropriate for data that reflect the behaviour of neuronal populations, such as EEG and fMRI data. The neural mass model can be viewed as a special case of ensemble density models, where the ensemble density is summarised with a single number representing mean activity [44]. Assuming that the equilibrium density of the neuronal states has a point mass (i.e., a delta function), we can reduce the density dynamics to the location of that mass. What we are left with is a set of non-linear differential equations describing the evolution of this mode.

The time variations of membrane potential in the individual compartments of the pyramidal cell and single compartment interneurons, 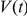, are determined by the differential equation for a simple voltage source circuit:
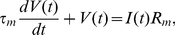
(1)where 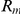 is the effective membrane resistance of the compartment, and is cell-type and compartment specific. 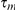 is the membrane time constant (same for all cells and compartments). The current, 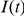, that flows through the membrane of the cell depends on the connections between different elements of the cortical unit and its external inputs (Figure 1(a)). The cortical unit receives external excitatory input in different subpopulations, whilst its sole output is the firing rate of the pyramidal cells, 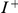. The excitatory inputs to the transmission interneuron, 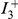, and basal dendrites of the pyramidal cell, 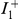, correspond to thalamo-cortical afferent projections. The input to the apical tuft dendrites, 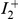, mediates cortico-cortical interactions. These currents can be found in Figure 1(a).

In terms of synaptic connections within the cortical unit, the total inhibitory synaptic effect on the pyramidal cell is given by: 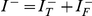, where 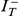 is the transmission inhibitory current and 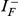 the feedback inhibitory current. The inhibitory synaptic currents depend nonlinearly on the membrane potential of the GABAergic cells through a threshold function: 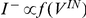. The excitatory synaptic current generated by the pyramidal cell has the same form: 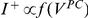:
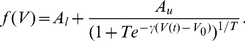
(2)


The parameters are set to 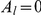 and 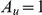 to ensure that the output stays between 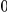 and 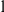. The 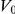 and 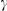 parameters determine the voltage sensitivity by setting the membrane potential maximum growth and growth rate, respectively. These parameters are estimated from the data. 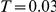 determines the membrane potential near the asymptote where maximum growth occurs. The threshold function, 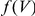, is also used to construct the firing rate coupling model (see below).

The equations for the membrane potential at the soma of the three-compartment pyramidal cell, as well as the extracellular potential along its apical dendrites can be determined from the potentials and currents at the individual compartments (given by Eq. 1). These equations can be found in Text S1. The apical dendrites of the layer V pyramidal cells are arranged in parallel to each other and perpendicularly oriented to the surface of the cortex. This geometry facilitates the summation of electric currents in the neuropil. The mesoscopic effect resulting from the spatial average of these extracellular currents corresponds to the electrical signal measured with EEG.

The state variables, 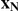, and parameters, 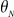, of the neural mass model described above are summarised in Table 3 of the main text and Tables 1 and 2 in Text S1.

**Table 3:** Estimated parameters: these are the parameters estimated from synthetic and measured EEG-fMRI data (one example session, all frequencies).

Electrical, vascular and coupling parameters							
					Synthetic		Observed
Type	Description	Symbol	Units	Prior	True	Estimated	Estimated
Electrical ( 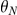 )							
	Synaptic input	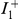	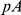	1.00	0.80	0.85	0.94
	Synaptic input	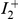	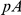	1.00	1.00	1.00	1.00
	Synaptic input	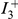	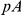	1.00	0.50	0.60	0.60
	GABAergic IN synaptic factor	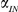	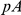	0.30	0.50	0.49	0.53
	PC voltage-ampere function	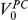	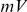	0.60	0.90	0.78	0.42
	V PC voltage-ampere function	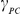	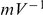	6.00	4.00	5.62	5.95
Vascular ( 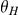 )							
	Signal decay	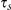	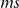	0.65	0.50	0.65	0.59
	Autoregulation	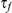	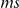	0.41	0.28	0.41	0.40
	Transit time	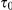	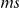	0.98	0.78	0.98	0.91
	Stiffness	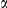	no dim.	0.32	0.25	0.32	0.32
	Resting O2 extraction fraction	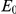	no dim.	0.34	0.30	0.34	0.34
Coupling							
NO model ( 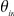 )	NO concentration baseline	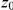	no dim.	0.10	0.30	0.29	0.29
	NO synaptic current factor (IN)	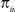	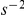	1.59e03	1.50e03	1.59e03	1.59e03
FR model ( 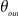 )	PC voltage-ampere function	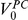	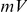	0.78	0.90	0.63	0.17
	PC voltage-ampere function	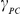	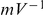	5.62	4.00	5.70	7.98
Mixture model ( 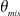 )	NO coefficient	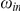	no dim.	0.50	0.40	0.40	0.29
	FR coefficient	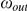	no dim.	0.50	0.60	0.60	0.71

The parameter 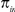 for the synaptic input model corresponds to: 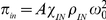 (see Eq. 6–8 and Table 2 in Text S1).

### Extended Balloon model

The coupling between local neuronal activity, described by the neural mass model, and subsequent changes in vascular dynamics is our question of interest. These changes are expressed in the BOLD signal and have previously been modelled in an extended Balloon approach [30], in which a set of four ordinary differential equations comprise the hemodynamic forward model from ‘neuronal activity’ to hemodynamic responses. The full derivation of these equations can be found in [29] and [30]. In brief, for a particular region, neuronal activity, 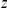, causes an increase in a vasodilatory signal, 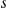, that is subject to auto-regulatory feedback. Inflow, 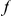 responds in proportion to this signal with concomitant changes in blood volume 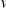 and deoxyhemoglobin content 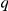. These equations are summarised in Text S1.

The hemodynamic parameters, 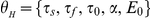, comprise the rate constant of the vasodilatory signal decay, the rate constant for autoregulatory feedback by blood flow, transit time, Grubb's vessel stiffness exponent, and the resting oxygen extraction fraction, respectively.

The whole dynamic system is driven by the input 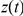. Different inputs, 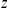, correspond to different aspects of neuronal activity and consequently different coupling hypotheses between neuronal activity and the BOLD response. A summary of the hemodynamic model's state variables, 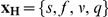, and parameters, 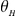, can be found in Table 3 of the main text and Tables 1 and 2 in Text S1.

In the next section we specify the neurovascular coupling mechanisms we are interested in comparing.

### Observation equations

The original electro-vascular model proposed by [36] is represented by a set of stochastic differential equations describing the dynamics of the neuronal and vascular states, 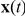. In [36] the stochastic aspect of the model is instantiated by incorporating an additive multidimensional Wiener process to model physiological noise. In this paper, however, we use a deterministic version of the model. This means that the dynamics are completely determined by the state of the system and stochastic effects enter only at the observation level (Eq. 3). This deterministic approach resulted in very similar frequency-response curves to those in [36] (see Results: synthetic data below) and allows us to use standard Bayesian estimation routines, widely used with deterministic forward models for EEG (e.g. [42]) and fMRI (e.g. [40]).

The observation equations for EEG, 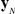, and fMRI, 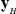, data are then given by:
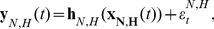
(3)where the errors are assumed to be i.i.d., 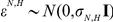.

The temporal variations of the EEG signal are well approximated by the extracellular electric current in the neuropil, 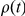, obtained from the NMM multiplied by the lead field matrix, 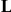. This matrix contains information about the geometry and conductivity of the head, and is therefore employed to map the distributed electric sources within the brain to scalp EEG recordings [45]:
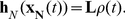
(4)


The observation function for fMRI is a static nonlinear function of the cerebral blood volume and the concentration of deoxyhemoglobin directly [30]:
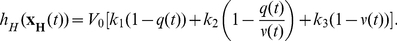
(5)


The factors 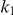, 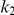 and 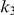 are dimensionless but depend on the characteristics of the fMRI recording system. For 1.5 T and TE of 40 msec, 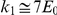 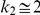 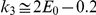. 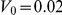 is the resting blood volume fraction.

### Neurovascular coupling

To link the two main components of the biophysical model, the neural mass model and the Balloon model, we specified three different biologically plausible neurovascular coupling mechanisms based on previous empirical results. These mechanisms are described below:

#### Synaptic input model

The first model considered assumes that the input to the Balloon model, 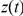, depends on the amount of nitric oxide (NO) released by synaptic activity, as originally proposed by [36]. We refer to this model as the *synaptic input model*.

NO is a potent vasoactive and rapidly diffusing gas [46], being a good candidate for regulating blood flow during functional activation [13], [47]. Although its synthesis is not yet fully understood, neuronal NO is thought to be generated pre-synaptically [12] and increases in NO concentration have been reported following increases in synaptic activity [48].

The total concentration of NO in the cortical unit is modelled as a nonlinear function, 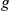, of the transmembrane capacitive currents in the somas of the interneurons and of the pyramidal cell. Although the genesis of NO is thought to be pre-synaptic [36], assume a direct causal relation between pre-synaptic activity and changes in post-synaptic transmembrane currents. These currents can be obtained from the derivative of the membrane potential, 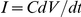, (see Eq. 1) and therefore the total concentration of NO is given by:
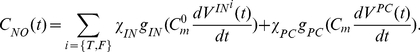
(6)


The energetic factors 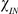 and 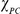 are introduced in order to make a distinction between relative metabolic demand in neurons of different types. 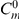 and 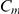 are the effective membrane capacitances in the somas of the neurons. To take into account both inward and outward ionic currents, the nonlinear function, 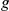, is required to be symmetric around zero and to include a saturation effect (Figure 1(b)):
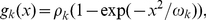
(7)where 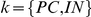 and 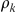 and 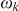 are parameters to be estimated from the data.

The amount of NO released in the cortical unit (Eq. 6) is then passed through a low-pass filter with gain 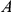, cut-off frequency 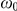 and damping factor 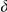. Finally, the input to the extended Balloon model, 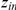 (and derivative 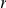), is given by:
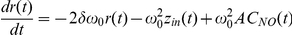


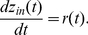
(8)


**Figure 2:**
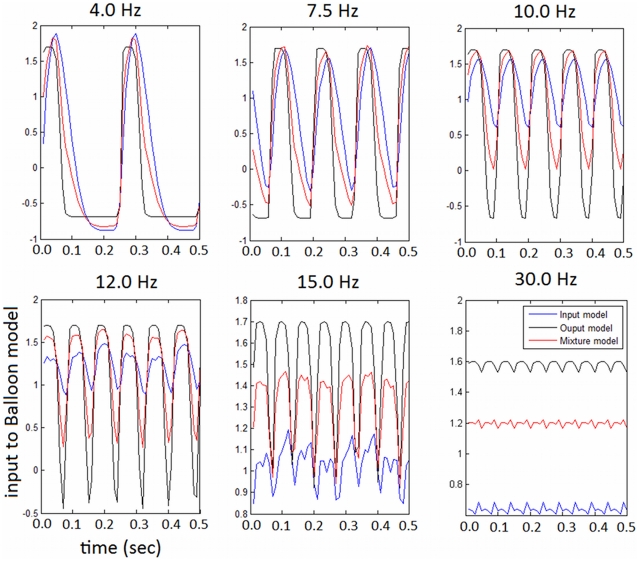
Input to Balloon model for different frequencies. Synaptic input model (blue), 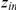, spiking output model (black), 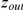, and mixture model (red), 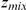. The signals have been standardised (mean corrected and divided by the standard deviation of the signal).

The baseline concentration of NO before stimulation, 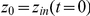, is estimated from the data. In total, this model has seven free parameters, 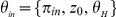, which are estimated from the data (Table 3). The time series of 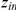 (input to the Balloon model) can be found in Figure 2 for most frequencies.

#### Spiking output model

For the second neurovascular coupling hypothesis we consider blood flow to be driven by the output spikes of the cortical unit, i.e the firing rate of the pyramidal cells. We refer to this model as the *spiking output model*.

The spiking activity of the layer V pyramidal cells is the outcome of the processing of information in the cortical unit and contains the information that is transmitted to other areas within and outside the cortex. Therefore this model looks at how BOLD signals are related to the output of local neuronal information processing as opposed to the synaptic input assessed by the previous model.

In this model the generalised logistic function (Eq. (2)) is employed to transform the average membrane potential of the pyramidal cell population, 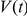, into the average rate of action potentials fired by these neurons [49] (Figure 1(c)):
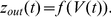
(9)


This model has seven free parameters (the same number of parameters of the input model), 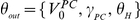, which are estimated from the data (Table 3). The time series of 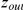 (input to the Balloon model) can be found in Figure 2 for most frequencies.

#### Mixture model

The third coupling model assumes that both synaptic and spiking activities can contribute to the generation of the hemodynamic signals. Therefore, the mixture model is a sum of the amount of NO released by synaptic activity in the cortical unit and the firing rate of its pyramidal cells:
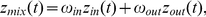
(10)where 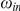 and 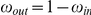 are coefficients to be estimated from the data and represent the relative contribution of each type of activity. This model has ten free parameters (three more parameters than the previous models), 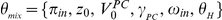 . The time series of 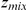 (input to the Balloon model) can be found in Figure 2 for most frequencies.

### EEG-fMRI data

#### Subjects and task

We use EEG and fMRI data from a previous study [34] to compare the neurovascular coupling models. In brief, the data were concurrently acquired using a synchronised acquisition protocol [50] for three healthy volunteers (three male, mean age 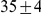 years exposed to visual flicker stimuli of varying frequencies. Three consecutive sessions of the same experimental task were recorded for each subject. A reversing black and white checkerboard (11×11 squares, size 13 cm×13 cm) was delivered via a computer monitor (60 Hz refresh rate) and projected on a screen positioned 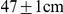 from a 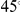 mirror located 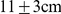 from the subject (visual angle 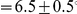). The reversing frequencies used were 4.0, 7.5, 10.0, 12.0, 15.0, 20.0 and 30.0 Hz. Stimuli were delivered in epochs of 5 scans (15.3 sec), followed by periods of 5 scans of rest (blank screen), and the order of stimulus blocks was randomised. Subjects were instructed to view a fixation cross which was visible during both rest and stimulus periods, and no overt response was required in either condition. The paradigm used here was designed to induce a large response in sensory cortex, in order to study a basic physiological mechanism, the neurovascular coupling. Although luminance levels were not held constant for the different flicker frequencies, these values were measured and taken into account by scaling the input to the model appropriately.

#### fMRI data

Images were acquired from a 1.5 T whole-body scanner (Magnetom Sonata, Siemens Medical, Erlangen, Germany) operated with its standard body transmit and circularly polarised head receive coil. The manufacturer's standard automatic 3D-shim procedure was performed at the beginning of each experiment. The scanner produced T2*-weighted images with a single-shot gradient-echo EPI sequence. Whole brain images consisting of 34 contiguous transverse slices, on a 64-by-64 grid, were acquired every 3.06 seconds resulting in a total of 320 functional scans for each of the three sessions of each subject (slice thickness = 2 mm, gap between slices = 1 mm, repetition time TR = 90 ms, flip angle = 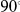, echo time TE = 50 ms, field of view 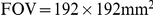, and therefore 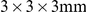 voxel resolution). Whole-brain structural scans were also acquired using a T1-weighted 3D-Modified Driven Equilibrium Fourier Transform (MDEFT) sequence [51] in 176 sagittal partitions with an image matrix of 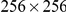 (TR = 12 ms, TE = 4 ms, flip angle = 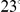, and voxel size 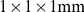).

The fMRI data were pre-processed with SPM8 software (http://www.fil.ion.ucl.ac.uk/spm/) implemented in Matlab (The Mathworks, Inc.). The first five scans of each session were discarded, and the pre-processing steps included: (a) realigning the images to the first scan and coregistering the structural scan with the mean functional image from all sessions; (b) correcting for differences in acquisition time between slices and normalising all the functional and structural scans to a standard EPI template based on the Montreal Neurological Institute (MNI) reference brain in Talairach space [52] (c) smoothing the functional images (Gaussian kernel, 8 mm half width). The movement parameters obtained from the realignment step were included in the subsequent general linear model (GLM) analysis as confounding covariates. The data were also high-pass filtered with a cut off period of 128 sec, to remove scanner drift and physiological noise.

**Figure 3:**
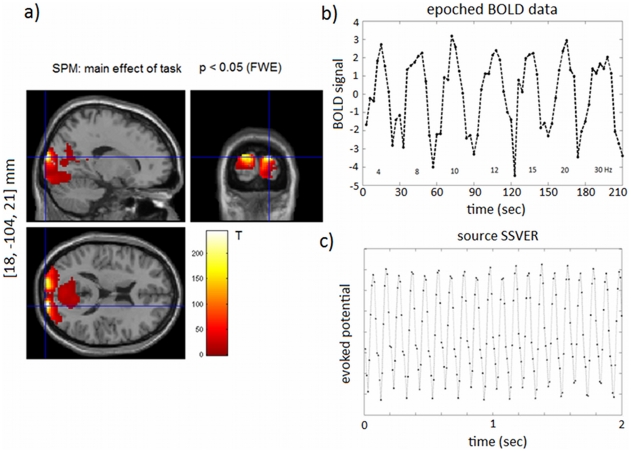
EEG-fMRI data. a) SPM results (3 sessions, example subject): effect of visual flicker stimulation on fMRI data. The voxel location corresponds to the most significant cluster maximum (Talairach space), 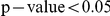 (FWE). b) Epoched BOLD signal (eigenvariate) from the most significant cluster maximum - one example session. c) 2 second source SSVER, 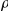, from the same cluster peak from 1 example session and frequency (10 Hz). Both signals have been standardised (mean corrected and divided by the standard deviation of the signal) as used in the optimisation scheme.

In previous work [34] we identified the brain regions activated by the flickering checkerboard in each subject. These regions are located in the subjects' visual cortex, as expected (see Figure 3(a) for an example subject). The coordinates of the corresponding cluster maxima are: 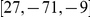 mm, 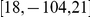 mm and 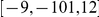 mm (Talairach coordinates). From these location we extracted the BOLD signal (200 scans per session) by calculating the first principal component of the adjusted data (removing the global drift and other confounds) from voxels within a 6 mm spherical volume centered on the cluster maximum. The resulting time-series for each session were then epoched and averaged (in the time domain) across epochs (Figure 3(b)). These time-series were used to estimate the parameters of the neurovascular coupling model, as described below.

#### EEG data

EEG was acquired with an MR-compatible BrainAmp amplifier and BrainCap EEG cap with ring Ag/AgCl electrodes (Brainproducts GmbH, Munich, Germany). Raw EEG was sampled at 5 kHz and a low pass filter (cut off frequency: 1 kHz) was used. This system provided 29 EEG channels, 2 EOG channels, and 1 ECG channel. The electrodes were distributed according to the 10/20 system, and the reference electrode was located between Fz and Cz. We additionally measured the pulse using a pulse oxymeter attached to the subject's finger and the locations of the EEG electrodes were recorded with a Polhemus digitiser.

The EEG data were pre-processed as described in [34]. The data acquired inside the scanner were corrected off-line using facilities in the Brain Vision Analyzer software package (Brainproducts GmbH, Munich, Germany) [53]. The gradient artefact was removed via mean subtraction with template drift compensation, whilst cardiac related artefacts were removed by subtracting the first three principal components that were time-locked to pulse oxymeter readings. The data were then high-pass filtered (0.5 Hz) to reduce slow drifts in the signal. The quality of the data acquired inside the scanner was assessed by comparing it to the data acquired outside the MR-environment, as described in [34]. In addition, electrodes Fp1 and Fp2 were discarded due to eye-blink artefacts.

Here we use the scalp steady state visual evoked responses (SSVERs) to reconstruct the electrical activity at the source level. SSVERs were computed by first epoching the artefact-corrected 27-electrode EEG data acquired inside the MRI scanner, for each session, in a 15-second post-stimulus window and then averaging (in the time domain) across trials. This procedure yielded 7 averaged 15-second time-series for each session corresponding to the 7 different flicker frequencies used. The source electrical activity was then obtained as follows. Given a source region with known anatomical location, we can form the 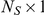 lead field vector 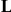 where 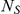 is the number of EEG sensors. This vector was obtained with SPM8 using a template mesh for the location and orientation of the cortical source and a boundary element method for the head model. The source location was chosen to be the corresponding cluster maximum identified with the fMRI data (see previous section). Given that the number of sources (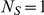) is smaller than the number of scalp channels (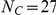), activity in the source region can be estimated as follows [54]:
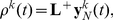
(11)where 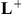 denotes the Moore-Penrose pseudo-inverse of the lead field vector 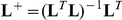. Here 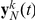 is the artifact-free SSVER for frequency 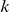 and one session. The resulting source time-series (for all frequencies and all sessions), 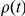, were used to estimate the parameters of the neural mass model (see below) (Figure 3(c)).

### Bayesian model inversion

Using EEG-fMRI data in combination with Bayesian inference allows us to estimate the underlying synaptic and spiking activities, along with other parameters of the biophysical framework. Additionally, we can compare the different neurovascular coupling hypotheses using Bayesian model evidence.

In Bayesian inference, prior beliefs about parameters, 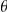, of model 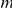 are quantified by the prior density, 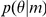. Inference on the parameters, 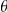, after observing data, 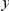, is based on the posterior density 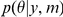. These densities are related through Bayes' rule:
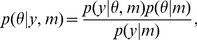
(12)where 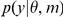 is the probability of the data (likelihood) conditioned upon the model and its parameters. The normalisation factor, 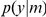, is called the model evidence and plays a central role in model comparison (see below).

The posterior density is an optimal combination of prior knowledge and new observations, weighted by their relative precision (i.e., inverse variance), and provides a complete description of uncertainty about the parameters. Generally, the choice of priors reflects either empirical knowledge (e.g., previous measurements) or formal considerations (e.g., biological or physical constraints). Here we use empirical knowledge for both the neural mass model parameters and the coupling/hemodynamic parameters, based on estimates obtained by [36].

Under Gaussian assumptions, also known as a fixed-form Laplace approximation [55], the problem of estimating the posterior density reduces to finding its first two moments, the conditional mean 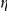 and conditional covariance 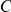. The prior density is also assumed to be Gaussian with mean 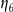 and covariance 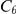 (see Table 3 for a list of prior mean values).

A non-linear model, such as the local electro-vascular (LEV) model used here, Eq. (3), can be linearised by expanding the observation equation about a working estimate 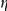 of the conditional mean:
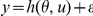


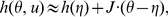
(13)such that 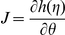, 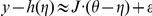 and 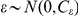. In this paper, the error covariance is assumed isotropic over the EEG and fMRI predictions 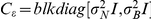.

The linearised model, Eq. (13), can be used in a Variational Laplace (VL) optimisation scheme that iteratively updates the moments of the conditional density, 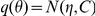. VL is a generic approach to estimate the posterior density, and can be formulated by analogy with statistical physics as a gradient ascent on the ‘negative Free Energy’, 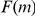, of the system. The full derivation of the algorithm is described in [56].

The maximisation of 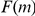 with respect to 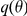 in effect maximises a lower bound on the log model evidence, 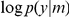, [57]:
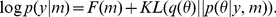
(14)


The model evidence is the probability of obtaining observed data, 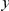, given model, 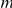, and is at the heart of Bayesian Model Selection (BMS). The last term in Eq. (14) is the Kullback-Leibler (KL) divergence between the approximate posterior density, 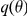, and the true posterior, 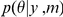. This quantity is always positive, or zero when the densities are identical, and therefore 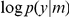 is bounded below by 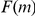. Through the iterative optimisation described above, the KL divergence is implicitly minimised and 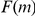 becomes an increasingly tighter lower bound on the log-model evidence. Model comparison can then proceed using 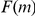 as a surrogate for the log-model evidence.

This approximation to the posterior density has been evaluated using Markov Chain Monte Carlo (MCMC) [58]. These schemes are more computationally intensive but allow one to estimate the posterior density without assuming it has a fixed form. Comparison between the model evidence obtained by MCMC methods and by variational approaches showed similar estimates, confirming that the approximations entailed by the variational approach lead to accurate model selection [55].

#### Multi-step inversion

The use of both EEG and fMRI data to estimate the electro-vascular model is affected by the difficult problem of how to deal with the disparity between the two datasets' time scales. In our study, for each fMRI point (sampled every 3 secs) we have 300 EEG data points (sampled at 100 Hz). The large amount of EEG data renders the model inversion computationally intensive, as for each parameter update we must integrate the model equations at a fine temporal scale (1000 Hz).

To overcome this problem we developed a computationally efficient inversion scheme based on partitioning model inversion into separate steps depending on the time-scales of the data involved. We refer to this scheme as a ‘multi-step inversion’ approach. This procedure generalises to other datasets and can be used in other multimodal studies, such as MEG-fMRI or LFP-fMRI, where the amount of data and time scales are very different between modalities.

**Figure 4:**
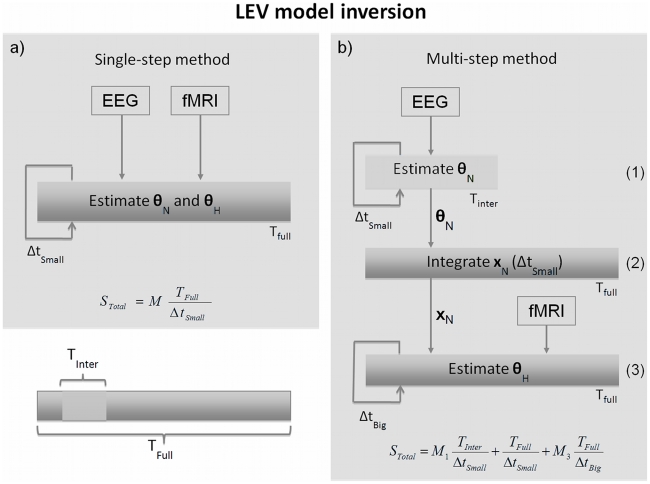
LEV model inversion. Here we adopted a ‘multi-step’ approach as opposed to inverting the model in a single step. a) Single-step approach: the EEG and fMRI data are used to estimate the neuronal and hemodynamic parameters (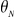 and 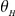) simultaneously. At each iteration the model equations are integrated at a small time scale matching that of neuronal activity, 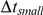, for the entire time interval, 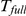. b) Multi-step method: here the inversion is performed in three main steps. (1) First the neuronal parameters, 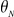, are estimated (using 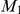 iterations) from the EEG data with a fine temporal resolution, 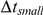, but for a smaller period, 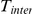 (2 seconds). (2) In the second step these parameter estimates are used to integrate the neuronal equations of the LEV model, 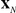, with the same temporal resolution 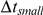 but entire time interval 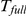. (3) In the last step we use the BOLD data to estimate (using 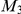 iterations) only the hemodynamic parameters, 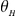, with a lower time resolution of 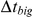 over the full time interval, 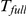. The total number of time steps, 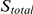, for each approach is displayed in each gray box.

This ‘multi-step inversion’ approach works as follows (Figure 4):

1. First we selected 2 secs of the source SSVERs (Eq. 11) for each frequency (4 to 30 Hz) and session to identify the electrical states, 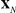, and parameters, 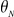 of the NMM. Using the EEG data alone to estimate the parameters of the NMM makes sense because these data are not dependent on the changes in the vasculature that give rise to BOLD. We chose to fit only 2 secs for each frequency (concatenated and chosen from the middle of the stimulation block to avoid onset and offset transients) because, as reported in [38], the averaged signal for the entire 15 secs is very regular (stationary), being sufficient in our view to estimate the model without using the entire trial block (Figure 4). Reducing the data to 2 secs per frequency considerably speeds up the inversion process. The parameters for each session were estimated iteratively using a time step of 1 msec. At each iteration the predictions were downsampled by a factor of 10 in order to fit the 100 Hz source SSVER data. Here we assume the neuronal response is stationary within a given epoch (15 sec stimulus interval) with averaged EEG and BOLD signals used here.
2. After estimating the electrical parameters (previous step), we used these estimates to integrate the full LEV model. Importantly, this integration takes place only once (as opposed to a ‘single-step’ approach, where it would have to be integrated at every iteration). The integration is implemented as above but instead of 2 secs, the input to the model is now 15 secs of stimulation and 15 secs of rest for each frequency. We integrate the full models with the three different coupling mechanisms described above and produced the following time-series as our input to the BOLD response (next step). For the synaptic input model the output time-series is the total NO concentration, Eq. (8). For the spiking output model the output time-series is the firing rate of pyramidal cells, Eq. (9), whilst for the mixture model both of these output time-series were produced, Eq. (10). These output time-series were downsampled to 10 Hz to reduce the estimation time of the next step and used as inputs to the Balloon model.
3. Finally, with the time-series for all coupling models obtained in the previous step we estimated the extended Balloon model using the epoched BOLD data for all frequencies. The estimation was again performed iteratively as described above (Figure 4), this time with a 100 msec time step because the vascular dynamics is a much slower process than the electrical processes. The value of the free energy (surrogate to the log model evidence) for each neurovascular model was then used to infer the optimal coupling mechanism.

### Bayesian model selection

Again through Bayes' rule we can relate the model evidence to the model posterior probability, 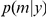:
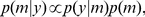
(15)where 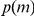 is the prior distribution over models. Selecting the optimal model corresponds to choosing the model 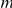 that maximises the posterior 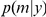. If no model is favoured *a priori* then 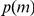 is a uniform distribution, and the model with the highest posterior probability is also the model with the highest evidence, 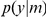.

Given two models, 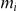 and 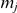, we can compare these models using Bayes Factors, 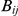 [59], which are defined as the ratio of the corresponding model evidences, or equivalently the difference in their log-evidences:
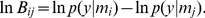
(16)


Bayes factors have been stratified into different ranges deemed to correspond to different strengths of evidence. ‘Strong’ evidence, for example, corresponds to a BF of over 20 (log-BF over 3) [59] in favour of model 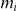 when compared to model 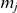. The equivalent posterior model probability is greater than 0.95 [43]. Here we use Bayes factors to compare the neurovascular coupling models defined in the previous section.

## Results

### Synthetic data

In this section, simulations are used to explore the behaviour of the model and its ability to reproduce EEG and BOLD data under the experimental conditions described in the previous section. The response of the three neurovascular coupling models to changes in stimulus frequency is also shown. These synthetic signals are used to test the model inversion routines and to verify that Bayesian model comparison can be used to infer the correct coupling model.

The LEV model was numerically integrated using the multi-step Adams-Bashforth-Moulton predictor-corrector algorithm implemented in the MATLAB (The MathWorks, Inc.) function *ode113*. The integration step used was 1 msec (1000 Hz) for the electrical and vascular states. The integrated signals were then downsampled to 100 Hz in the EEG case and to 0.3 Hz for the BOLD signal. The input to the model is described below.

#### Model input

The input to the LEV model was generated by creating a series of single events with the same frequency as the reversing checkerboard (4.0, 7.5, 10.0 … Hz). These events are modelled as Gaussian functions of 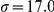 msec width: 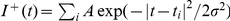. This value of 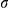 corresponds to the screen refresh interval. The amplitudes 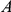 are fixed over time but differ for excitatory versus inhibitory populations. In our simulations we used the amplitudes 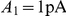 and 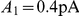 for 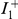 and 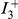, respectively, as proposed in [36]. These amplitudes are estimated from the data when using the EEG-fMRI signals (see below). Input 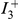 was also delayed by 100 msec with respect to 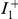 as suggested in [36]. Cortico-cortical interactions were neglected and so 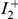 was set to zero during the entire period of integration. Due to the fact that luminance levels were not kept constant for the different frequencies we multiplied the input time-series according to the lux measures (from low to high frequencies) by: 1.00, 0.96, 0.93, 0.91, 0.88, 0.82, 0.74 (lower frequencies had higher luminance levels).

#### Frequency-response curves

**Figure 5:**
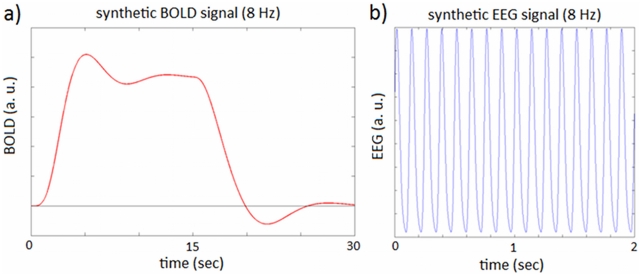
Simulated data. a) BOLD response for a stimulation block (15 seconds of stimulation and 15 second of rest) of 8 Hz reversing frequency; b) EEG signal for the same stimulus (2 seconds). Both signals have been standardised (mean corrected and divided by the standard deviation of the signal) as used for model inversion.

We first generated data from the LEV model separately for the different stimulus frequencies (4 to 30 Hz). We used the three neurovascular coupling mechanisms described above. The data were simulated using the parameter values summarised in Table 3 and Table 2 (Text S1) for a period of 15 seconds of stimulation and 15 seconds of rest. The simulated signals showed that all coupling models predict an increase of the BOLD signal during stimulation, as expected, and synchronisation of the EEG signal to the input frequency. Figure 5 shows the EEG and fMRI signals generated for a period of 15 sec of stimulation and 15 sec of rest using the synaptic input model.

**Figure 6:**
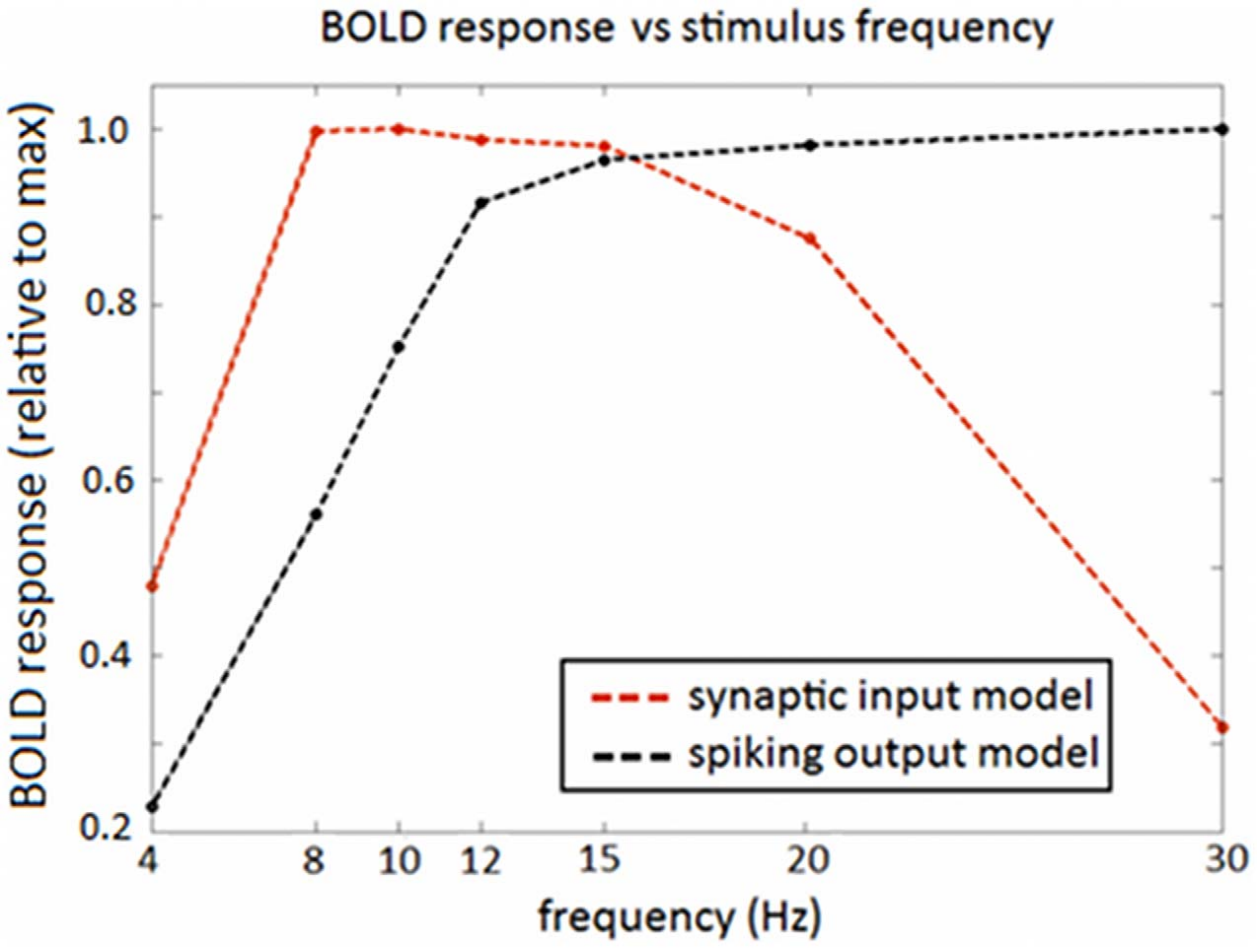
Model frequency response curves -synthetic data. a) Predicted BOLD response versus reversing frequency for the synaptic input and spiking output models. The curves show the BOLD response obtained for each stimulus frequency (divided by the maximum peak for each model).

We then looked at the behaviour of the fMRI signal predicted by the different coupling models for all frequencies. Figure 6 presents the frequency-response curves obtained. These curves correspond to the maximum amplitude of the BOLD signal for each stimulus frequency. As can be seen in Figure 6, the synaptic input model predicts an increase in the BOLD response until approximately 8 Hz and a decrease afterward. This result confirms the simulations of [36] who found a similar frequency-response curve for the NO mechanism between (0.5 and 16 Hz). In addition, this result validates the use of a deterministic model instead of the original stochastic model. The stochastic effects are therefore not necessary to reproduce the frequency response curve obtained in [36]. Contrary to the synaptic input model, the spiking output model predicts an increase in the BOLD response with input frequency without any saturation effect (Figure 6).

**Figure 7:**
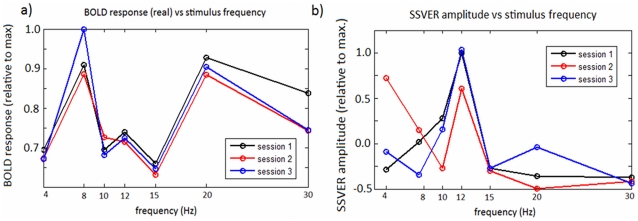
Measured frequency response curves - EEG-fMRI data. a) Measured BOLD response versus reversing frequency. The values on the y-axis correspond to per cent changes of the global mean signal. b) Frequency-response curve for EEG data. Each point corresponds to the amplitude of the evoked response (divided by the maximum response) at that frequency (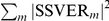). The maximum value was 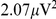.

Figure 7(a) shows the frequency-response curve for the real fMRI data. For real data the values plotted in this curve correspond to GLM coefficients as a function of frequency (stimulus). These are obtained when we regress the BOLD signal using the onsets of the stimuli as our regressors, or columns of the design matrix. Each column corresponds to a different frequency and the associated coefficient tells us how much BOLD is expected to increase with that particular frequency. As can be seen in Figure 7(a), the response of the real BOLD signal to the different frequencies also peaks at 8 Hz and has a minimum at 15 Hz. This behaviour has been previously reported in human BOLD data for frequencies below 16 Hz under similar experimental conditions [60]–[62]. Above 15 Hz this curve has a second peak in BOLD signal amplitude at 20 Hz and a decrease afterward (Figure 7(a)). The same type of curve is reported in [61]: two maxima at 8 and 20 Hz, a smaller peak at 12 Hz, and the rest of the frequencies (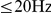 in [61]) lie below these values.

The frequency-response curve for the measured SSVERs is plotted in Figure 7(b). The curves for all three sessions of an example subject show a peak at 12 Hz and a decrease in amplitude afterward. This same curve was found in all other subjects and sessions. This means the peaks in the BOLD signal cannot be explained from the electrical signals alone.

#### Model parameters

Table 3 lists the parameters for the electric, 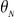 and vascular, 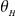, components of the model that are estimated from the data. These are the same parameters estimated in [38]. We also summarise the coupling parameters in the same table: 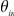, 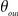 and 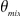 (Table 3). The amplitudes of the three input currents (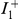, 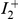 and 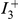) and 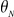 are estimated from EEG in step (1) of the inversion. 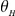 are estimated from the BOLD signal in step (3). 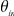 and 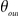 and 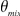 are estimated from both EEG and fMRI data in steps (1) and (3) of model inversion.

When using the observed EEG and fMRI signals, the priors on the parameters corresponded to the parameter estimates obtained by [38], that is, from the inversion of the same electro-vascular model with similar EEG-fMRI data. Prior variances were chosen to be of the same order of magnitude as the prior means to ensure a coefficient of variance (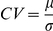) of approximately 1 for all parameters.

#### Model comparison

We then tested if Bayesian model comparison could be used to correctly decide upon which coupling model was used to generate the data, and if despite the small number of samples of fMRI compared to EEG we could still infer the right model.

We again generated data using the three coupling models as described above. We generated data for all the frequencies concatenated, with additive Gaussian observation noise: 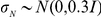 and 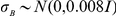. These values are based on the signal-to-noise ratio for the observed data (1 for the averaged EEG signals and 2 for the averaged BOLD signals). We then fitted the coupling models to each of the three synthetic datasets.

**Figure 8:**
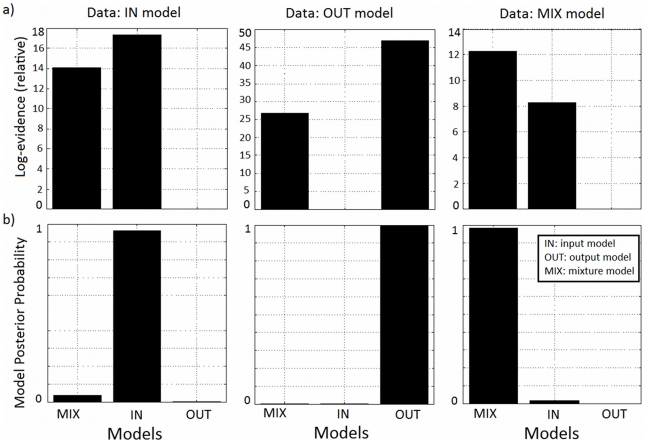
Model comparison with synthetic data. We generated data with the different coupling models (IN: synaptic input model; OUT: spiking output model; MIX: mixture model). We then fitted these datasets with the same three coupling models and obtained the results plotted in the figure. a) Difference in log-evidences relative to worst model. b) Corresponding model posterior probabilities.

We verified that Bayesian model comparison inferred the correct model in all cases, with a minimum Bayes factor of approximately 20 (log-Bayes factor of 3) (Figure 8). This value corresponds to strong evidence in favour of the model that generated the data and a posterior model probability over 0.95 [43]. The parameter values used to generate the data and the corresponding parameter estimates and priors for each model can be found in Table 3. As can be seen the parameter estimates were close to the real values used in data generation.

As an aside, we note that, as with any gradient-ascent based optimisation algorithms, our inversion scheme is subjected to the possibility of running into local minima. However, one way to tackle this problem can be to initialise the inversion in different parameter regimes. In this work we have only observed once a clear case of local minimum, where the fit of one of the models to one session was extremely poor. We have then initialised the parameters with the estimates from other sessions and the inversion scheme was able to find new parameter estimates that provided a good fit to the data, similar to what was obtained for the other sessions.

### EEG-fMRI data

**Figure 9:**
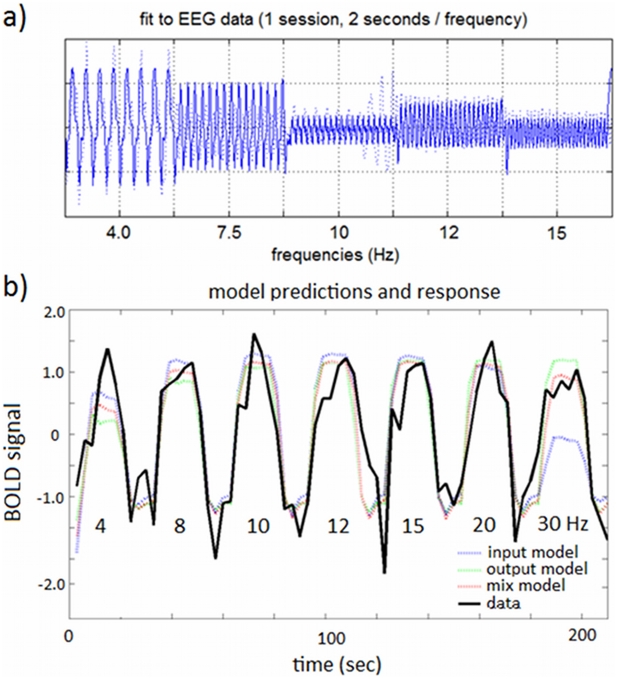
Model identification from EEG-fMRI data. a) EEG time-series (dotted line) and model fit (solid line) for one example session and subject (2 seconds of data per frequency). b) Model predictions and BOLD data for the same example session and subject (all frequencies: 4 to 30 Hz). As can be seen in the figure, the input model (blue) provides the best fit to the BOLD data (black) for the lowest frequencies (e.g. 4.0 and 7.5 Hz), whilst for the highest frequency (30 Hz) it's clear that this model underestimates the BOLD response. The output model (green) provides a better fit for this frequency but predicts a higher response than the one observed. The signals have been standardised (mean centred and divided by the standard deviation of the signal) as used in the model inversion scheme.

Finally we fit the electro-vascular model with the three different coupling mechanisms to the EEG and fMRI data. We used the same ‘multi-step’ inversion procedure described in the previous section. Figure 9 shows the model predictions for EEG, as well as predictions of the coupling models and the BOLD response.

#### Model comparison

**Figure 10:**
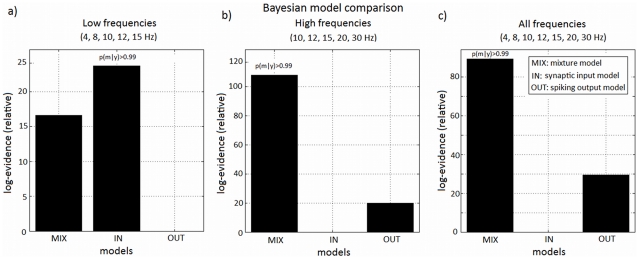
Model comparison. (MIX: mixture model; IN: synaptic input model; OUT: spiking output model): log-model evidence relative to worst model (for low, high and all frequencies). These are group results for all subjects and sessions analysed (the log-evidences are summed over subjects).

Our analysis focused on the relevant contributions of synaptic and spiking activity models as a function of stimulation frequency. To this end we divided the stimuli into ‘low-frequencies’ (4 to 15 Hz), ‘high-frequencies’ (10 to 30 Hz) and ‘all-frequencies’ (4 to 30 Hz) and the analysis was repeated for these three regimes. A summary of the model comparison results for all subjects can be found in Figure 10. The results for all sessions, subjects and frequency regimes can be found in Table 3 of Text S1.

As can be seen in Figure 10(a), in the low-frequency regime we found that the synaptic input model best explained the observed data. In this regime the spiking output model was the worst model. The difference in log-model evidence between the best model (synaptic input) and the second best model (mixture) was above 5. This value corresponds to strong evidence in favour of the synaptic model and a probability, 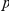, over 0.99 of this model being the best model to explain the data in this regime [43]. This result was consistent accross subjects and sessions analysed (5 out of 6 sessions) (Table 3 of Text S1). The model evidence values can be found in Table 3 in Text S1.

However, when we analysed the high frequencies, the mixture model was found to be the best model with probability 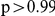 (Figure 10(b)). This result was again consistent across subjects and for the majority of sessions (7 out of 9 sessions) (Table 3 of Text S1). In this regime the spiking output model was the second best and, contrary to the low-frequencies case, synaptic activity contributed the least to the BOLD response.

For both regimes, the inferred neuronal firing rates were found to be commensurate with the stimulation frequency. Finally, an additional analysis across all frequencies revealed that the mixture model was the best model, again with probability 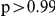 (Figure 10(c)). This result was found in 6 of the 9 sessions analysed, although in one of the sessions the model evidence for all three models was nearly identical (Table 3 of Text S1).

We note here that it has come as no surprise the fact that when we analyse all frequencies the mixture model was found to explain the data better than the input and output models alone. As we observe in Figure 7(a), the double peaked frequency-curve of fMRI data can be easily explained by a weighted combination of the frequency-response curves predicted for the input and output models individually (Figure 6). This weighted combination is the definition of the mixture model and the weights (mixture parameters) depend on the regime of frequencies analysed, providing, for instance, a one-peaked or two-peaked curve for low and all-frequencies, respectively.

These results were robust to the choice of partition into low/high frequencies. Similar results (not shown) were obtained with partitions such as: low-frequencies (4, 8, 10, 12 Hz) and high-frequencies (15, 20, 30 Hz).

## Discussion

In this paper we used EEG-fMRI data and a biophysically informed mathematical model to investigate the relationship between neuronal activity and the BOLD signal in human visual cortex. In particular, we explored the contributions of synaptic input and spiking output activities to the generation of the BOLD response.

We have provided preliminary evidence that the BOLD signal is dependent upon both synaptic and spiking activity but that the relative contribution of these two factors are dependent upon the underlying neuronal firing rate. When the underlying neuronal firing is low then BOLD signals are best explained by synaptic input, in agreement with previous animal studies, such as [3]. This result is also in line with more recent studies, such as [9] and [10], which show that the BOLD response is only affected by changes in synaptic-related activity (measured with LFPs) and not by changes in spiking activity (measured with MUA) when these two signals can be dissociated.

However, when the neuronal firing rate is high then both synaptic and spiking activity are required to explain the BOLD signal, as observed in, for example [6], and [15]. We were particularly encouraged to find that a combination of synaptic input and spiking output frequency response curves (Figure 6) can explain the doubly-peaked BOLD response observed by [6] and replicated in our own data.

One possible explanation for the increased performance of the output model with higher frequencies comes from neuroenergetic studies such as e.g. [7] and [63]. In these studies brain metabolism was found to depend strongly on neuronal spiking, with increases in oxygen consumption reflecting higher firing rates. More recently [64], have found that differences in the BOLD response between different brain areas (motor cortex and thalamus) could be explained by underlying differences in the firing rates of the corresponding neuronal populations.

Our results also support the conclusion that the relationship between synaptic activity, spikes and BOLD signals depends on the specific neuronal circuitry engaged in task processing. Moreover, one can speculate that different coupling mechanisms involving different types of cells and molecules could come into play depending on the task in question.

Despite our initial concern about the small number of fMRI samples compared to EEG, our initial results with synthetic data showed that it is possible to make inferences on different hypotheses for the neurovascular coupling using a generative modelling framework and Bayesian model comparison. The issue of different time-scales was addressed by partitioning the estimation of electrical and vascular states into a multi-step approach. In this approach we first estimated the electrical states and parameters from the EEG data and then integrated the full electro-vascular model using these estimates. From the integrated model we extracted the input time-series to the Balloon model, which we then inverted using BOLD data. The last two steps were repeated for each coupling model.

This method significantly increases the computational efficiency of the model inversion. However, this multi-step approach is only possible with a deterministic model. In this work we used a deterministic version of the stochastic electro-vascular model proposed by [36]. Under different experimental conditions, which do not induce a large sensory response, the introduction of stochastic effects might be essential to reproduce the empirical data. In this case, other Bayesian inversion frameworks can be employed to estimate the model parameters, such as [65] and [66].

It is also worth noting that despite the fact that the mixture model had more parameters than the input and output models, this extra complexity did not provide a significantly better fit to the data in the low-frequency analysis than the input model. This complexity is correctly penalised using Bayesian methods, such as the one used here.

One concern about the coupling models defined here regards the definition of NO concentration. As mentioned in the Methods section, NO is thought to have a pre-synaptic synthesis [12], [13]. However, here and in [36] the concentration of NO is modelled through post-synaptic quantities such as the transmembrane capacitive currents. Although in principle these two phenomena are directly related (increases in pre-synaptic activity mean larger post-synaptic effects) this is not always the case. Changes in transmembrane currents at the post-synaptic level can be caused by different processes such as chemical-gated channels, electric-gated channels, and passive leakage, not all of them being related to pre-synaptic activity. Therefore the transmembrane currents are an indirect way of quantifying the amount of NO released during synaptic activity. However, this issue is also encountered in experimental measures of synaptic activity, such as local field potentials. This signal is a surrogate post-synaptic signal, which is also affected by other slow potentials occurring at the cellular level that do not have a purely pre-synaptic origin.

A natural extension to this work is the inclusion of multiple cortical units in the model representing multiple brain areas. For instance, sub-cortical areas such as the thalamus and other cortical areas activated by the experimental task could be included. Having more than one area would facilitate the differentiation between input and local processing synaptic activity, such as in [37]. In a recent study [67], have decomposed the effect of these two types of synaptic activity on hemodynamic signals by reducing the thalamic input to a rodent's cortex. The authors found that although both input and local neuronal processing contribute to BOLD signals, as previously found, this contribution is larger from local processing.

Another extension would be to probe the contribution of excitatory and inhibitory neuronal populations to the generation of BOLD signals, such as in [39]. This model-driven approach could, for instance, be used to study the findings of [68], where a negative BOLD response in deeper cortical layers, adjacent to positive-BOLD areas, was found to be associated with a reduction in local neuronal firing. Very recently [64], have optically driven genetically modified inhibitory cells and measured a negative BOLD signal in response to this stimulation, in the rat cortex. This result can inform the development of new generative models of neurovascular coupling.

To our knowledge this paper presents the first quantitative model comparison of different biologically plausible mechanisms for neurovascular coupling in human cortex using EEG-fMRI data and a realistic biophysical model.

However, even though our results were consistent across the three subjects and the majority of sessions, the case study approach adopted here has its limitations. Namely, it does not quantitatively address the issue of inter-subject variability and it therefore precludes inferences at the population level. With a larger sample of subjects, inter-subject variability can be accommodated using the Random-Effects (RFX) model selection approach developed by [69]. This approach fits a Bayesian hierarchical model to group model evidence data to obtain the frequencies with which each model is used in the population. This approach can be combined with the methodology developed in this paper. We hope that future studies with other datasets and different experimental conditions will employ our modeling approach so that a balance of evidence can be reached that clearly disambiguates between different hypotheses concerning neurovascular coupling.

Understanding the underlying biophysical mechanisms behind the coupling between neuronal activity and the BOLD response is vital not only for improving the interpretability of the BOLD response, but also for relating findings from fMRI research with results from other neuroscientific disciplines.

## Supporting Information

Text S1 We present the full biophysical model (i.e. all the equations that comprise the neural mass model and Balloon model used in this work, as well as their parameter values). We also provide detailed results of model comparisons for all subjects and sessions.
(PDF)
