# Supplementary material for: Bayesian Comparison of Neurovascular Coupling Models Using EEG-fMRI
Source: PLoS Comput Biol. 2011 Jun 16;7(6):e1002070. doi: 10.1371/journal.pcbi.1002070 (PMC3116890; doi:10.1371/journal.pcbi.1002070)
Supplement: Text S1 — We present the full biophysical model (i.e. all the equations that comprise the neural mass model and Balloon model used in this work, as well as their parameter values). We also provide detailed results of model comparisons for all subjects and sessions. (PDF) [file pcbi.1002070.s001.pdf]

Supporting material

S.1 Electrovascular model

Neural mass model The equations used to generate the electrical dynamics in the cortical unit are summarised below:

$$
\begin{aligned}
\tau_m \frac{dV_{IN}^T(t)}{dt} + V_{IN}^T(t) &= I_3^+(t) R_m^0 \\
\tau_m \frac{dV_{IN}^F(t)}{dt} + V_{IN}^F(t) &= I^+(t) R_m^0 \\
\tau_m \frac{d\nu_1(t)}{dt} + \nu_1(t) &= R_m^1 I_1^+(t) \\
\tau_m \frac{d\nu_2(t)}{dt} + \nu_2(t) &= R_m^2 I_2^+(t) \\
\tau_m \frac{d\nu_-(t)}{dt} + \nu_-(t) &= R_m I^-(t) \\
\tau_m \frac{dV_{PC}(t)}{dt} &= -(\alpha_0 + \sum_k \frac{1}{\beta_k}) V_{PC}(t) + \frac{\Omega(t)}{\prod_k \beta_k} + R_m I^-(t) \\
&+ \sum_k \left[\frac{R_m \nu_k(t)}{(R_i^k + R_e^k)} - \frac{\nu_-(t)}{\beta_k} \right] \qquad (1) \\
\tau_m \frac{d\Omega(t)}{dt} &= R_m \sum_k \frac{\beta_k (V_{PC}(t) - \nu_k(t))}{(R_i^k + R_e^k)} + V_{PC}(t) + \nu_-(t) - \Omega(t) \\
\tau_m \frac{d\rho(t)}{dt} &= -(\alpha_0 + \sum_k \frac{1}{\beta_k}) \rho(t) + \frac{\Theta(t)}{\prod_k \beta_k} \\
&+ \frac{R_e^2}{(R_i^2 + R_e^2)} (R_m I^-(t) + R_m^2 I_2^+(t)) \\
&+ \frac{[R_m^1(\nu_-(t) + \nu_2(t)) + R_m(\nu_2(t) - \nu_1(t))] R_e^2}{\prod_k (R_i^k + R_e^k)} \\
\tau_m \frac{d\Theta(t)}{dt} &= \left[1 + R_m \sum_k \frac{1}{R_m^k} \right] \rho(t) - \Theta(t). \qquad (2)
\end{aligned}
$$

Balloon model Below is a summary of the Balloon model's equations:

$$
\begin{aligned}
\frac{ds(t)}{dt} &= z(t) - \frac{s(t)}{\tau_s} - \frac{f(t) - 1}{\tau_f} \\
\frac{df(t)}{dt} &= s(t) \\
\tau_0 \frac{d\nu(t)}{dt} &= f(t) - \nu(t)^{1/\alpha} \qquad (3) \\
\tau_0 \frac{dq(t)}{dt} &= \frac{f(t)}{E_0} [1 - (1 - E_0)^{1/f(t)}] - q(t) \nu(t)^{(1-\alpha)/\alpha}.
\end{aligned}
$$

Table 1. Electrical and vascular states

Electrical and vascular states			
Type	State	Symbol	Initial value
Electrical			
	Membrane potential at the soma of GABAergic IN (Transmission)	V_{IN}^{T}	0
	Membrane potential at the soma of GABAergic IN (Feedback)	V_{IN}^{F}	0
	Membrane potential at the soma of Layer V PC	V_{PC}	0
	Voltage difference	Ω	0
	Equivalent voltage source at the layer V PC basal dendrites	ν_1	0
	Equivalent voltage source at the layer V PC apical tuft dendrites	ν_2	0
	Equivalent voltage source at the soma of layer V PC	ν_-	0
	Extracellular voltage difference along the layer V PC apical tuft	ρ	0
	Voltage difference	Θ	0
	Time derivative of the input (extended Balloon approach)	r	0
	Input (extended Balloon approach)	z	z_0
Vascular			
	Flow-inducing signal	s	0
	CBF	f	1
	CBV	ν	1
	Concentration of dHb	q	1

Table 2. Fixed parameters: these parameters are not estimated from the data.

Fixed parameters				
Type	Symbol	Parameter	Dimension	Value
Electrical				
	c_m	Membrane capacitance	$\mu F/cm^2$	0.75
	τ_m	Membrane time constant	ms	30
	C_m^0	Effective membrane resistance: soma compartment (IN)	nF	6.81
	C_m	Effective membrane capacitance: soma compartment (PC)	nF	1.045
	R_m^0	Membrane resistance: soma compartment (IN)	$G\Omega$	4.082
	R_m	Membrane resistance: soma compartment (PC)	$G\Omega$	2.871
	R_m^1	Membrane resistance: basal compartment	$G\Omega$	0.222
	R_m^2	Membrane resistance: apical tuft compartment	$G\Omega$	0.667
	R_i^1	Membrane resistance: basal longitudinal (intracellular)	$G\Omega$	0.226
	R_e^1	Membrane resistance: basal longitudinal (extracellular)	$G\Omega$	0.272
	R_i^2	Membrane resistance: apical longitudinal (intracellular)	$G\Omega$	2.264
	R_e^2	Membrane resistance: apical longitudinal (extracellular)	$G\Omega$	2.716
	α_{PC}	Layer V PC synaptic factor	$pA(0 \sim 1.5)$	0.4
	γ_{IN}	GABAergic IN voltage-ampere relationships	mV^{-1}	5
	V_0^{IN}	GABAergic IN voltage-ampere relationships	mV	0.7
	β_1	Basal voltage divisor	no dim.	2.24
	β_2	Apical voltage divisor	no dim.	7.45
	α_0	Mixed coefficient	no dim.	7.32
Others				
	χ_{PC}	Layer V PC energetic factor	$nM(0 \sim 1)$	1.0
	χ_{IN}	GABAergic IN energetic factor	$nM(0 \sim 1)$	0.8
	ω_0	Low-pass filter: angular high cut frequency	Hz	$2\pi 8$
	δ	Low-pass filter: damping factor	no dim.	0.8
	A	Low-pass filter: gain	nM^{-1}	1.0
	ρ_{PC}	Layer V PC nonlinear function	no dim.	1.0
	ρ_{IN}	GABAergic IN nonlinear function	no dim.	1.0
	ω_{PC}	Layer V PC nonlinear function	$(nA)^2$	0.1091
	ω_{IN}	GABAergic IN nonlinear function	$(nA)^2$	0.0464

Table 3. Model comparison results. Log-evidence values for each model, session and subject (low-frequencies, high-frequencies and all-frequencies). 'Sum' is the sum of the log-evidences per session and 'Group' the sum of all subjects' sums. The values in bold correspond to the winning model for each session and subject. BF_{12} are the log-Bayes factors between the best (1) and second best (2) model for each session. The values x^* correspond to a posterior probability for the best model higher than 0.95. The values x^{**} correspond to a posterior probability higher than 0.99. $^\diamond$One of the subjects lacked the 4.0 and 7.5 Hz stimulus epochs and was therefore not included in 'low-frequency' analyses.

Log-evidences, F_x, Bayes Factors B_{xy} and 'mixture' model coefficient (synaptic) ω_{in}						
Subject	Session	F_{mix}	F_{in}	F_{out}	BF_{12}	ω_{in}
Low	frequencies$^\diamond$	(4 to 15 Hz)				
1	1	-57.27	-55.69	-56.82	1.13	0.72
	2	-37.27	-34.89	-44.58	2.38	0.80
	3	-59.23	-59.01	-58.21	0.80	0.60
	Sum	-153.77	**-149.59**	-159.61	4.18*	0.71
2	1	-39.54	-38.53	-42.25	1.01	0.66
	2	-41.30	-40.16	-45.85	1.14	0.65
	3	-34.21	-33.40	-37.69	0.81	0.67
	Sum	-115.05	**-112.09**	-125.79	3.00*	0.69
Group		-268.82	**-261.68**	-285.40	7.14**	0.69
High	frequencies	(12 to 30 Hz)				
1	1	-37.85	-80.68	-43.22	5.37**	0.60
	2	-32.59	-45.90	-35.21	2.62	0.40
	3	-47.59	-44.56	-87.01	3.03*	0.83
	Sum	**-118.03**	-171.14	-165.44	53.11**	0.61
2	1	-69.48	-69.92	-68.57	0.91	0.55
	2	-47.24	-55.77	-84.73	8.23**	0.48
	3	-59.41	-68.00	-57.21	2.20	0.46
	Sum	**-176.13**	-193.69	-210.51	17.56**	0.50
3	1	-31.91	-43.06	-34.27	2.36	0.46
	2	-38.51	-49.64	-40.64	2.13	0.41
	3	-29.60	-44.52	-31.27	1.67	0.43
	Sum	**-99.72**	-137.22	-106.18	6.46**	0.43
Group		**-393.88**	-502.05	-482.13	88.25**	0.51
All	frequencies	(4 to 30 Hz)				
1	1	-37.85	-80.68	-43.22	5.37**	0.60
	2	-32.59	-45.90	-35.21	2.62	0.40
	3	-47.59	-44.56	-87.01	3.30*	0.83
	Sum	**-118.03**	-171.14	-165.44	53.11**	0.61
2	1	-89.78	-90.93	-88.10	1.68	0.56
	2	-71.94	-71.42	-71.56	0.14	0.65
	3	-79.46	-91.57	-80.48	1.02	0.59
	Sum	-241.18	-253.92	**-240.14**	1.04	0.60
3	1	-49.41	-61.05	-52.21	2.80	0.49
	2	-54.19	-64.21	-59.13	4.94*	0.46
	3	-44.94	-46.84	-50.76	1.90	0.58
	Sum	**-148.54**	-172.12	-162.10	13.56**	0.51
Group		**-507.75**	-597.18	-567.68	59.93**	0.57
